# A Real-Time Weed Mapping and Precision Herbicide Spraying System for Row Crops

**DOI:** 10.3390/s18124245

**Published:** 2018-12-03

**Authors:** Yanlei Xu, Zongmei Gao, Lav Khot, Xiaotian Meng, Qin Zhang

**Affiliations:** 1 College of Information and Technology, JiLin Agricultural University, Changchun 130118, China; mengxiaotian11@163.com; 2 Department of Biological Systems Engineering, Centre for Precision and Automated Agricultural Systems, Washington State University, Prosser, WA 99350, USA; zongmei.gao@wsu.edu (Z.G.); qinzhang@wsu.edu (Q.Z.)

**Keywords:** variable-rate herbicide spraying, weed map, particle swarm optimum algorithm, smart controller

## Abstract

This study developed and field tested an automated weed mapping and variable-rate herbicide spraying (VRHS) system for row crops. Weed detection was performed through a machine vision sub-system that used a custom threshold segmentation method, an improved particle swarm optimum (IPSO) algorithm, capable of segmenting the field images. The VRHS system also used a lateral histogram-based algorithm for fast extraction of weed maps. This was the basis for determining real-time herbicide application rates. The central processor of the VRHS system had high logic operation capacity, compared to the conventional controller-based systems. Custom developed monitoring system allowed real-time visualization of the spraying system functionalities. Integrated system performance was then evaluated through field experiments. The IPSO successfully segmented weeds within corn crop at seedling growth stage and reduced segmentation error rates to 0.1% from 7.1% of traditional particle swarm optimization algorithm. IPSO processing speed was 0.026 s/frame. The weed detection to chemical actuation response time of integrated system was 1.562 s. Overall, VRHS system met the real-time data processing and actuation requirements for its use in practical weed management applications.

## 1. Introduction

In agricultural crop production, indiscriminate application of herbicides leads to environmental pollution and reduced quality of the agricultural products. Variable-rate herbicide spraying (VRHS) technology can provide a solution to this problem as it can precisely adjust the application rates. It can lead towards minimal farmland pollution, safety of agricultural products and personnel, and sustainable development of agriculture [1,2]. 

The VRHS system can either use real-time machine vision to generate the weed map or use predetermined prescription charts [3,4]. The VRHS system based on prescription chart usually uses sensing in conjunction with Global Positioning System (GPS) and Geographic Information System (GIS) technology for the generation of prescription chart, from which the valuable data are obtained to determine the standard herbicide application rates, but the acquisition of prescription charts is very complex and there may exist large deviations from the real conditions. In addition, the prescription chart lacks real-timeliness [5,6]. However, conventional controller used in machine vision based VRHS system has very limited performance, logic operation speed, memory and communication capacity no matter what peripherals are used. This restricts the functionality of the VRHS system. 

The VRHS system based on a machine vision system uses Charge Coupled Device (CCD) camera inputs and image processing technology to control herbicide application rates. This technology is ideal for improving the utilization rate of herbicides [7,8]. However, the prerequisite is the fast and accurate extraction of weed distribution in the farmland to adjust the herbicide application rates [9]. Currently, different algorithms are being used to recognize the weed features [10,11,12,13,14,15]. Using Support Vector Machine (SVM), He et al. distinguished seedling stage maize from the weeds using a multiple morphological feature fusion method, with an accuracy of 95% [16]. Bai et al. identified field crops under different weather conditions using morphological modeling in Commission International de l’eclairage (CIE) color space [17]. Hernandez et al. established a non-parametric model, which identified farmland plants in color space using a probability density function [18]. Zhai et al. reconstructed the color model and recognized the crops in rapeseed images using a high pass Gauss algorithm with an accuracy of 98.2% [19]. Chen et al. extracted the color features of crops using the Cg component in YCgCr color space and a Simple Linear Iteration Cluster (SLIC) method, which overcame the problem of light variation in farm images [20]. Overall, the accuracy of some of the above algorithms was good, but the processing speed was a liming factor. 

There are many research institutions and scholars who have conducted relevant research on VRHS control systems [21,22]. The spraying system can be implemented mostly by adjusting the system pressure and pulse width modulation (PWM) to realize variable-rate spraying [23,24]. Regarding the pressure adjustment method, when the pressure in the spray nozzle changes (high or low) it inevitably affects the atomization at the fixed nozzle tip. However, the spray flow adjustment scope is limited. Also, although effective, the PWM adjustment method can lead to discontinuous spraying under low-frequency conditions [25,26]. Therefore, in order to resolve the above problems, the specific objectives of this study were:

(1) To develop a robust grayscale image segmentation algorithm-based weed mapping system.
(2) To integrate a variable-rate herbicide spraying (VRHS) system comprised of a grayscale imaging unit, data processing unit and multi-nozzle variable rate spray controller unit.
(3) To validate the field performance of our VRHS system towards applying chemicals on weeds distributed within a corn field with crop at the seedling growth stage.

## 2. Materials and Methods

### 2.1. Imaging System

Currently, a lot of weed mapping research is based on images collected by near infrared or multispectral cameras [27,28,29], which allow a simpler approach for the detection of vegetative material, but the main disadvantages of near infrared and multispectral cameras are the high initial investment and maintenance costs. Spectral crop sensors can easily cost some US $1000 [30]. Visible cameras are inexpensive and the technique is easy to use, even by unexperienced users. Moreover, visible cameras are suited for field operation and spectral cameras are more suitable for UAVs, so considering the actual conditions of our study region, this paper chose a visible camera.

The MV-EM040M industrial grayscale camera (Shaanxi Microvision Digital Image Technology Co., Ltd., Xi’an, China) was used to obtain farmland images. The camera has a spatial resolution of 1280 × 1060 pixels. The C-mount variable focus lens was chosen to go with this camera due to its good anti-shake attributes in capturing high-resolution grayscale images. The focal length of the camera was 4–10 mm. The aperture (F) was 1/1.4 and the field of vision (FOV) is 53.9°. The camera was mounted on top of a tractor, so several plant rows could be imaged at once. The spraying unit was assembled on back of the tractor, so there would be enough time to process the collected images.

**Figure 1:**
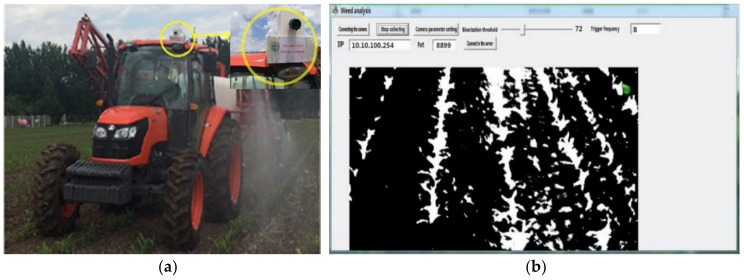
Imaging system used in study: (**a**) The variable-rate sprayer integrated imaging sensor (in yellow circle) for weed detection; (**b**) The imaging system interface.

During preliminary data collection, the speed of the tractor was maintained in the range of 2.5 m/s. The distance between the camera and the ground was 2.7 m. Corn plant images at the seedling stage (3–4 leaves stage) were collected on different sampling dates (four different days from 25 May to 10 June in 2017) and under sunlight conditions. Each time 50 corn plant images were collected, so the total number of images was 200. The heights of the corn and weeds were between 25–35 cm. The weeds have a tendency of cluster at this stage and be distributed unevenly, so it was suitable to implement variable-rate spraying. Then, the images were transferred to a computer by a network cable and processed to extract the weed map. A custom algorithm was developed on the Microsoft Foundation Class (MFC) application framework based on Microsoft Visual C++6.0 for this purpose. Figure 1 is the imaging system used in this paper, which is mounted on tractor. 

### 2.2. Segmentation Algorithm

#### 2.2.1. Particle Swarm Optimum Algorithms

Among threshold segmentation methods, the most popular method has been a maximum variance between clusters, also named Otsu’s method [31,32]. It divides images into target and background regions by setting a threshold and then calculates the average gray variance by traversing the whole thresholds. The threshold of largest variance is the best threshold value. Such an algorithm has good stability and robustness for operating on crop images and other kinds of images. Another advantage of OTSU is it is not affected by the light intensity. However, the speed of OTSU cannot meet the real-time requirement because the search for the optimal threshold needs to traverse the whole threshold range [33].

The particle swarm optimum (PSO) algorithm can obtain the threshold value quickly by reducing the number of iterations. The PSO algorithm simulates the foraging behavior of birds, and searches for the optimal value in complex space using information exchange and competition among individuals [34,35]. A PSO can quickly determine the maximum variance after several iterative optimizations within the image threshold, thus avoiding traversing the entire gray level [36,37]. However, the number of iterations will increase when the scale of the particle group is too large due to the fixed grayscale range, while the optimal rate of the algorithm cannot be guaranteed if the scale is too small. Thus, it is necessary to have an algorithm that resolves the above problems. Therefore, an improved particle swarm optimum algorithm (IPSO) was proposed and implemented in this study.

#### 2.2.2. Improved of Particle Swarm Optimum Algorithm

In this study, a new search strategy called improved particle swarm optimum algorithm (IPSO) was implemented. The method quickly searches for the global optimal particle by reasonable convergence of the particle swarm search scope. Then the small range of quadratic optimization can be performed by optimal particle, which improves the optimization rate of algorithm and calculation time. The specific operations of the IPSO algorithms are summarized in following steps:

(1) Calculate the average gray value of farmland images:
  (1) $$\mu_{L}\;=\;\sum_{i\;=\;0}^{L-1}n_{i}/\left(M\times N\right)$$
  where, *μ_L_* is the average gray value of farmland image, and the range of grayscale is [0, *L*]. The size of image is *M* × *N* and *n*_i_ represents the number of pixels of grayscale *i*.
(2) Search the maximum gray value *I*_max_ and the minimum gray value of *I*_min_ of the farmland image.
(3) Divide the farmland image into two regions called *A* and *B* by division value *μ_L_*. The ranges are *A*[*I*_min_, μ_L_], *B*[*μ*_L_, *I*_max_], respectively. The average gray values *μ*_A_, *μ*_B_ of *A* and *B* are calculated respectively:
  (2) $$\mu_{A}\;=\;\sum_{i\;=\;I_{\min}}^{\mu_{L}}n_{i}/\left(M\times N\right)$$
  (3) $$\mu_{B}\;=\;\sum_{\mu_{L}}^{i\;=\;I_{\max}}n_{i}/\left(M\times N\right)$$
(4) Set the search range of particle swarm in the range of [*μ*_A_, *μ*_B_].
  Particle swarm is initialized, and the particle size is set. The number of particle iteration and the random initial position and speed of particle are set as well. The particle swarm scale is updated:
  (4) $$G_{f}\;=\;\frac{\mu_{B}-\mu_{A}}{M\times N}\times G_{s}$$
  where, *G_f_* is the actual particle swarm size, *G_s_* is the initial setting of particle swarm size.
(6) Inter-class variance of OTSU function is used as the fitness of particle swarm, and the maximum variance of particle swarm is updated to obtain the optimal location of local particle swarm:
  (5) $$\sigma^{2}\;=\;\mu_{B}\cdot \mu_{A}{\left(I_{max}-I_{min}\right)}^{2}$$
(7) Particle swarm optimization algorithm can be expressed by Equations (6) and (7). The particle swarm position and speed can be adjusted for the next iteration according to Equations (6) and (7):
  (6) $$V_{iN}\left(t+1\right)\;=\;wv_{iN}\left(t\right)+c_{1}r_{1}\left(t\right)\left[p_{iN}\left(t\right)-x_{iN}\left(t\right)\right]+c_{2}r_{2}\left(t\right)\left[p_{gN}\left(t\right)-x_{iN}\left(t\right)\right]$$
  (7) $$x_{iN}\left(t+1\right)\;=\;x_{iN}\left(t\right)+v_{iN}\left(t+1\right)$$
  where, Equations (6) and (7) show that in an *N*-dimensional space, the space position of the *i*th particle is *X_i_* = (*x*_1_, *x*_2_, …, *x_N_*) (*i* is positive integer); *P_i_* = (*p_i_*_1_, *p_i_*_2_, …, *p_iN_*) is the optimal position that the particles traversed; *P_g_* = (*p_g_*_1_, *p_g_*_2_, …, *p_gN_*) is the optimal location of searching the whole particle swarm; the flight velocity of each particle is *V_i_* = (*v_i_*_1_, *v_i_*_2_, …, *v_iN_*); *t* stands for current evolutionary algebra; *c*_1_ and *c*_2_ represent the learning factor (acceleration coefficient); *r*_1_ and *r*_2_ are a random number distributed in [0, 1]; and *W* is the inertial weight.
(8) Algorithm iteration terminates when the algorithm reaches the maximum number of iterations or at search of the optimal adaptive value of the particle. Then the optimal global variable of the particle would be obtained. Otherwise it would return to formula (6).
(9) With the optimal particle location as the center, a search area with a range of *P* is set and scanned. The global optimum is updated when the particle with better fitness exists. Otherwise, the result will remain the same.
(10) Particle location of the optimal segmentation value is used as the optimum threshold to extract the farmland image.

**Figure 2:**
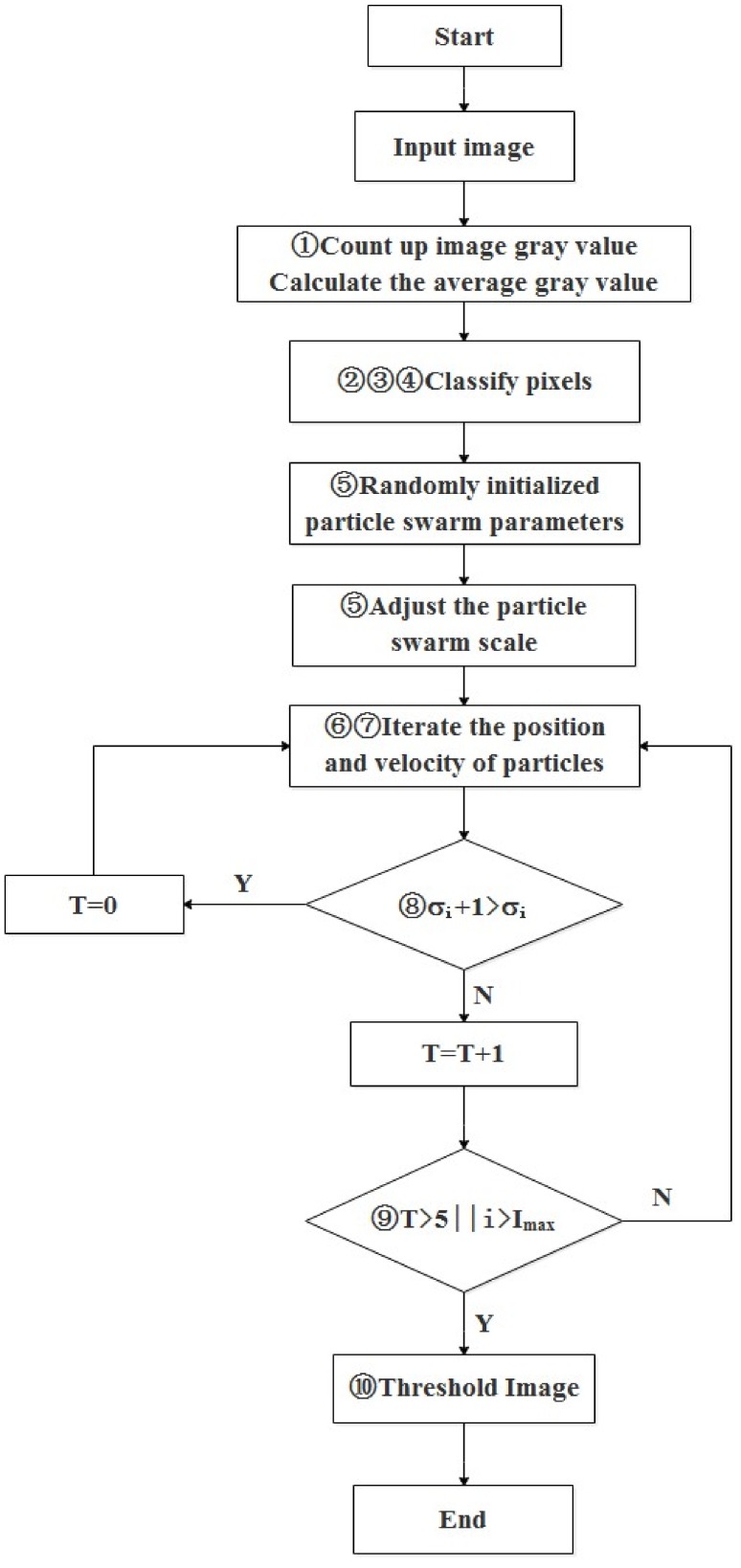
Flow chart of improved particle swarm optimum algorithm.

The flow chart of the IPSO algorithm is shown in Figure 2.

### 2.3. Weed Distribution Map

**Figure 3:**
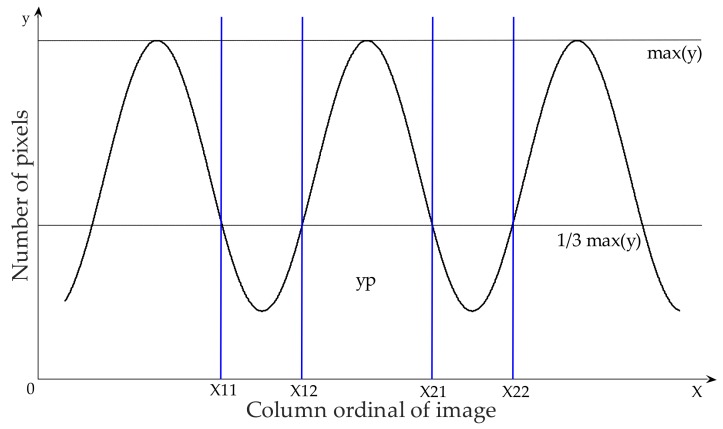
Schematic diagram of pixel lateral histogram.

The weed distribution information was extracted using an algorithm based on a position histogram. After the segmentation, the image was converted into a binary format by setting the background pixel value as 0 and crops and weeds as 1. Then the number of pixels with a value of 1 in each column of the image (*y*-axis of binary image) was counted, the lateral histogram of pixel distribution was obtained, as shown in Figure 3. 

The *x* axis represented column ordinal of the image, and *y* axis the number of pixels with value of 1 in the corresponding column. The maximum value of *y* was expressed as max(*y*). Setting *yp* ≤ 1/3×max(*y*), 1/3 is chosen based on specific situation and modification can be made if necessary, corresponding for the values of point *x*, several intervals of *x* are obtained, i.e., [*x*_11_ *x*_12_], [*x*_21_ *x*_22_], …, [*x_n_*_1_ *x_n_*_2_]. These intervals represent the actual spacing between the crops. For each interval ([*x_n_*_1_ *x_n_*_2_]), summation was performed for all values of *yp* (number of pixels). Thus, the sums of number of pixel values within the interval were expressed as *sum* (1), *sum* (2), *sum* (3), …, *sum* (*n*):

(8) p(n) = sum(n)/image size

where, *image size* represents the size of the image, i.e., total number of pixels; *p*(*n*) is the percentage of each inter-row weed pixel to total pixels of the image, i.e., the distribution percentage of inter-row weed. If the value of the *p*(*n*) is higher, that means more weed exists and then more herbicide need to be sprayed. According to common agricultural knowledge, the weed distribution intensity is divided into six levels: no weeds, few weeds, moderate weeds, many weeds, severe weeds, very severe weeds. The decision-making information was transmitted to the DSP controller via the Wi-Fi module for the variable-rate herbicide spray application.

### 2.4. Variable-Rate Herbicide Spraying Control System

The multi-nozzle VRHS system collected and processed the weed information and determined the herbicide application rates. The DSP controller received the signals and implemented the adjustment via the solenoid valves on the nozzles. The designed control system can achieve real-time adjustment of herbicide application rates and simultaneously monitor the liquid level, pressure and flow rate of the sprayer. The spraying control system encompassed three parts including spraying sub-system, monitoring unit, and the DSP controller.

#### 2.4.1. Multi-Nozzle Spraying Sub-System

**Figure 4:**
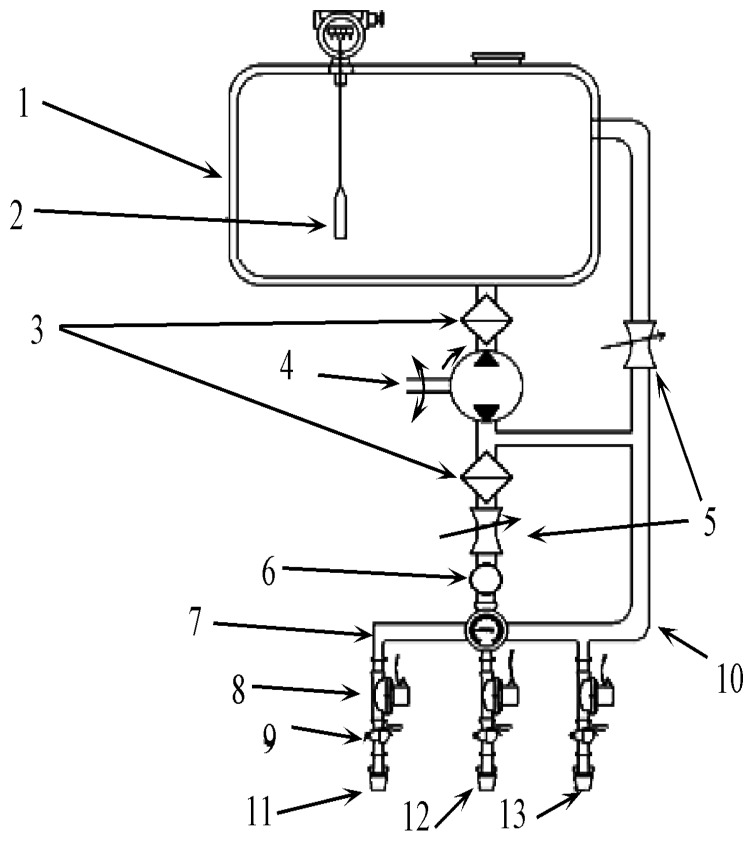
Multi-nozzle variable-rate spraying sub-system of the VRHS control system. 1. Herbicide tank; 2. Liquid level sensor; 3. Filter; 4. Diaphragm pump; 5. Safety valve; 6. Pressure sensor; 7. Distributor; 8. Solenoid valve; 9. Hall Flowmetre; 10. Backflow connection; 11. First drip-proof nozzle; 12. Second drip-proof nozzle; and 13. Third drip-proof nozzle.

The herbicide spraying sub-system comprised the herbicide tank, filter, diaphragm pump, safety valves, distributors, anti-drip nozzles and associated conduits (Figure 4). Before spraying, the herbicide was first mixed with water in the herbicide tank at a certain proportion. The diaphragm pump was driven by a transmission belt connected to the output shaft on the tractor. The diaphragm pump had two output channels. The herbicide would be returned to the tank via the safety valve circuit in one channel. In another channel, the herbicide passed through the filter and the safety valve and finally entered the spraying conduits, which branched into outlet pipe and bypass reflex pipe. The latter related to the herbicide tank via the safety valve circuit. The former was connected to the distributors, and each distributor was equipped with three anti-drip nozzles. The diaphragm pump would be driven by the tractor, then the herbicides would be sprayed onto the weeds. 

The VRHS control system has multiple nozzles, with three nozzles on each row. Each nozzle was equipped with a high-performance solenoid valve (2W160-15, SunHua, JiNing, China). Three types of Teejet nozzles were used, namely, fan-shaped XR series XR11001VS, XR11002VS, and XR11003VS. The spray cone angle was 110° uniformly and the nozzles could achieve open directional spraying. Thus, all seven modes of variable-rate herbicide spraying would be achieved with the nozzles including mode-1 (nozzle 1 open), mode-2 (nozzle 2 open), mode-3 (nozzle 3 open), mode-4 (nozzle 1 and nozzle 2 open), mode-5 (nozzle 1 and nozzle 3 open), mode-6 (nozzle 1 open), mode-7 (nozzle 1, nozzle 2 and nozzle 3 open). 

#### 2.4.2. Monitoring Unit

A monitoring unit was designed for the VRHS control system. A Hall flow meter sensor (YF-B3, ZhongJiang JieNeng, FoShan, China), liquid level sensor (HD-136, HuaDian Automation, BeiJing, China) and pressure sensor (HY-131-A, HengYun Instrument, TianChang, China) were installed in-line as shown in Figure 4 (9, 2, 6). When herbicide was sprayed with different combinations of opening and closure of solenoid valves, the liquid crystal display (LCD) would show the current working state of the system. 

The liquid level sensor in the herbicide tank monitored the herbicide level. The pressure sensor was installed in front of the distributor. This would help to indicate whether the pipelines were blocked or whether a fault occurred to the distributor in real-time. Three of the YF-B3 Hall flow meters were installed below the three solenoid valves, respectively, to monitor the flow rates in the three nozzles.

#### 2.4.3. Variable-Rate Herbicide Spraying Control System

**Figure 5:**
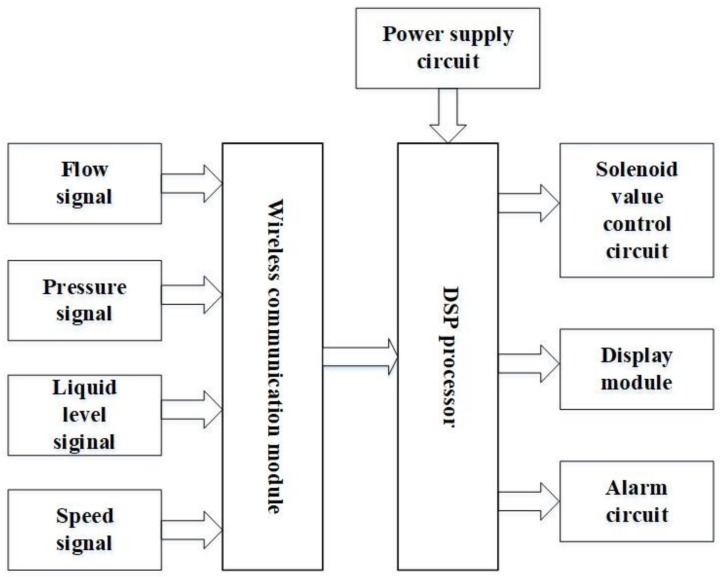
Block diagram of variable-rate herbicide spraying controller implemented in this study.

The block diagram of the VRHS controller operational flow is shown in Figure 5. The wireless communication module included a USR-C322 WiFi Module, level converter circuit and reset circuit constitute, which was responsible for the data transmission between the computer and controller. The solenoid valve control circuit was composed of relay (HK4100F, HuiKe, ShenZhen, China) and associated drive circuit. DSP controlled the opening and closure of the relay, with a result controlling the opening and closure of multiple nozzles. The system circuit of DSP comprised DSP chip (TMS320F2812, Texas Instruments, Dallas, TX, USA) and the peripheral circuit. DSP controller received the signals of herbicide application rate from the computer and then adjusted the flow rate of the nozzles. The controller also received and processed feed from sensors on the working state to be displayed on the monitoring unit. The signal acquisition circuit collected the signals of flow rate, pressure, speed, and fluid level. These signals were transmitted to the DSP controller via the wireless communication module. The power supply circuit provided DC current for all circuits. The alarm circuit would trigger the buzzer and LED circuit when the pipelines were blocked, the spraying pressure exceeded the normal constant value, or the fluid level in the herbicide tank was too low. Thereby, the operator would be informed and start to do the repair work immediately. 

Once VHSR system switched-on, the image collection module would collect images in the field and generate the weed map. If weeds were not detected or the amount of weeds detected did not reach the set threshold for decision-making, the computer would return to the previous loop without sending actuation signals. In addition, the relay circuit would be disconnected when the DSP controller did not receive the signals from the computer. As a result, the solenoid valves would not be actuated. If weeds are detected, the computer would send weed distribution information, which was transmitted via the wireless communication module to the DSP controller. Once the decision-making information was received, the DSP controller would send signals to turn on or off the relay, thus realizing different combinations of opening and closure of the solenoid valves. During this loop, the signals of fluid level of herbicide in tank, flow rate of three nozzles, pressure readings from the distributors and operating speed of the spraying machine would be transmitted to the DSP processor via the wireless communication module in real-time. Such information would be displayed on the LCD for monitoring purposes.

### 2.5. Field Validation Experiment 

**Figure 6:**
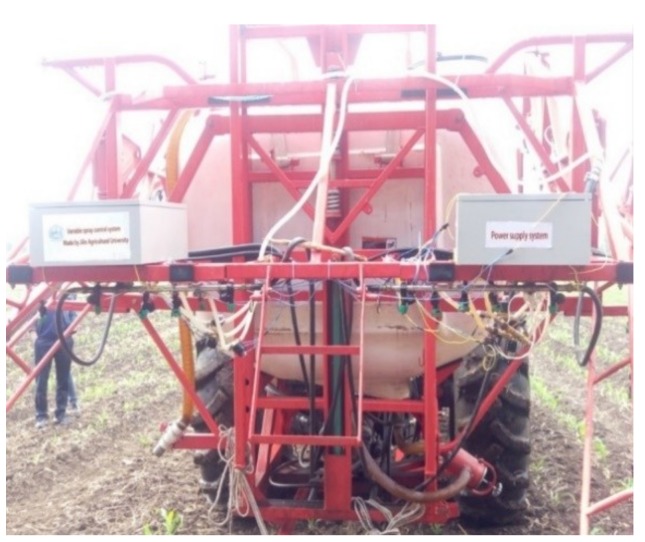
Field testing of the variable-rate spraying system.

To validate the feasibility and validity of the weed mapping system and VRHS control system, the integrated system was installed on a spraying tractor. The imaging camera was put in front of the tractor, as shown in Figure 1a. Images were acquired to extract the weed distribution and hence determined the herbicide application rate, as shown in Figure 1b. The field validation was implemented in the testing field of JiLin Agricultural University, Changchun, China. The spraying system can obtain variable spraying data for three rows, as shown in Figure 6.

During the experimental test, the self-propelled herbicide spraying machine would move at a uniform speed of 2.5 m/s. The running speed of the program not only depended on the optimization degree of the algorithm, but also the hardware performance, memory space and CPU running frequency, which would affect the processing speed to some extent. The time required to process an image and send spraying instruction was thus calculated, using the “time. h” function, to evaluate the integrated system performance. The central processor was configured with an Intel Core i3-3220 3.30 GHz CPU and 2 GB memory.

The time that the spraying system received the signal and implement spraying operation can be calculated as well. It depended on the processor’s speed, the signal delay and the mechanical delay. Furthermore, the real-time processing of whole VRHS system was validated by field experiment. 

## 3. Results and Discussion

### 3.1. Image Segmentation 

**Figure 7:**
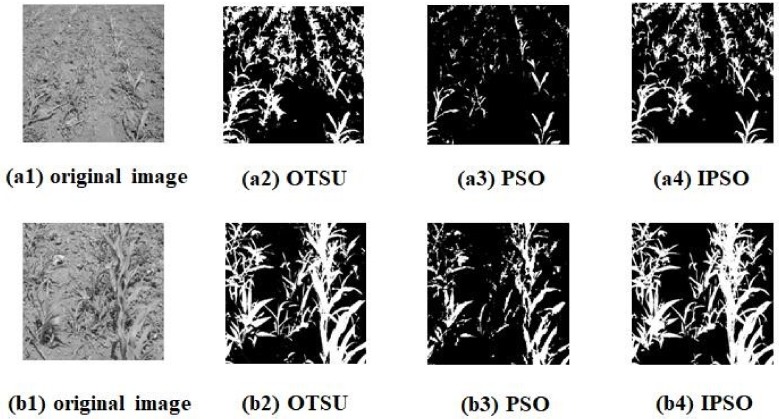
Output images of segmentation using different algorithms: (**a1**,**b1**) Original images collected by sensor; (**a2**,**b2**) Segmentation images using traditional OTSU algorithm; (**a3**,**b3**) Segmentation images using PSO algorithm; (**a4**,**b4**) Segmentation images using IPSO algorithm.

In this study, 30 farmland images collected from field experiments were segmented using traditional OTSU, PSO and IPSO, respectively. The particle swarm parameters were set as follows: number of original particle *M* = 15; the maximum number of iterations was 30; the learning factor *c*_1_ = *c*_2_ = 2; the inertia weight *w*_max_ = 0.8, *w*_min_ = 0.4. Figure 7 shows the segmentation results of two farmland images from thirty images, in which IPSO method has almost the same good processing effect as the OSTU method, while the PSO method result was not clear and lost some details. In order to evaluate the performances of the segmentation methods, the run time and optimization number of each algorithm were tested and counted. Then the optimization rate can be calculated with Equation (9) and the standard error rate was also defined by Equation (10):

(9) $$p\;=\;\frac{n}{N}\times 100\%$$

(10) $$\sigma \;=\;\sqrt{\frac{\sum_{i\;=\;1}^{N}{\left(T_{i}-TU_{i}\right)}^{2}}{N}}\;=\;\sqrt{\frac{\sum_{i\;=\;1}^{N}E_{i}^{2}}{N}}$$

where *n* was the number of optimizations and *N* was the total number of images (*N* = 30). *T*_i_ is the threshold value of different algorithms, and *TU_i_* is the threshold value of the OTSU method. *E_i_* is the difference of *T*_i_ and *TU_i_*. Table 1 lists the performances of the three segmentation methods, in which the threshold value was the mean value of 30 images.

As shown in Table 1, the OTSU method can obtain the optimal solution, but it was time consuming as it has to iterate through all grey scales to determine the optimal threshold. In addition, OTSU cannot realize real-time processing, while the speed of the PSO algorithm was fast. However, the segmented images were not clear and lost some details and hence the optimization rate was low. Compared with the standard PSO algorithm, the IPSO algorithm iterated more times, the computing time was close to PSO, but it improved the error rate of the optimal solution. With close efficiency, the IPSO algorithm was more accurate.

### 3.2. Weed Distribution Assessment 

**Figure 8:**
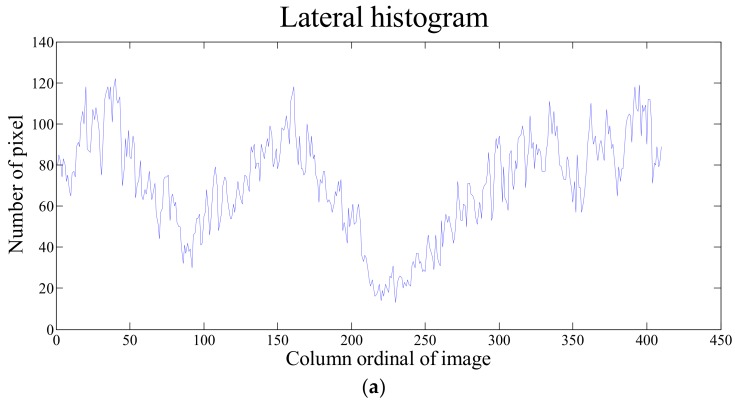

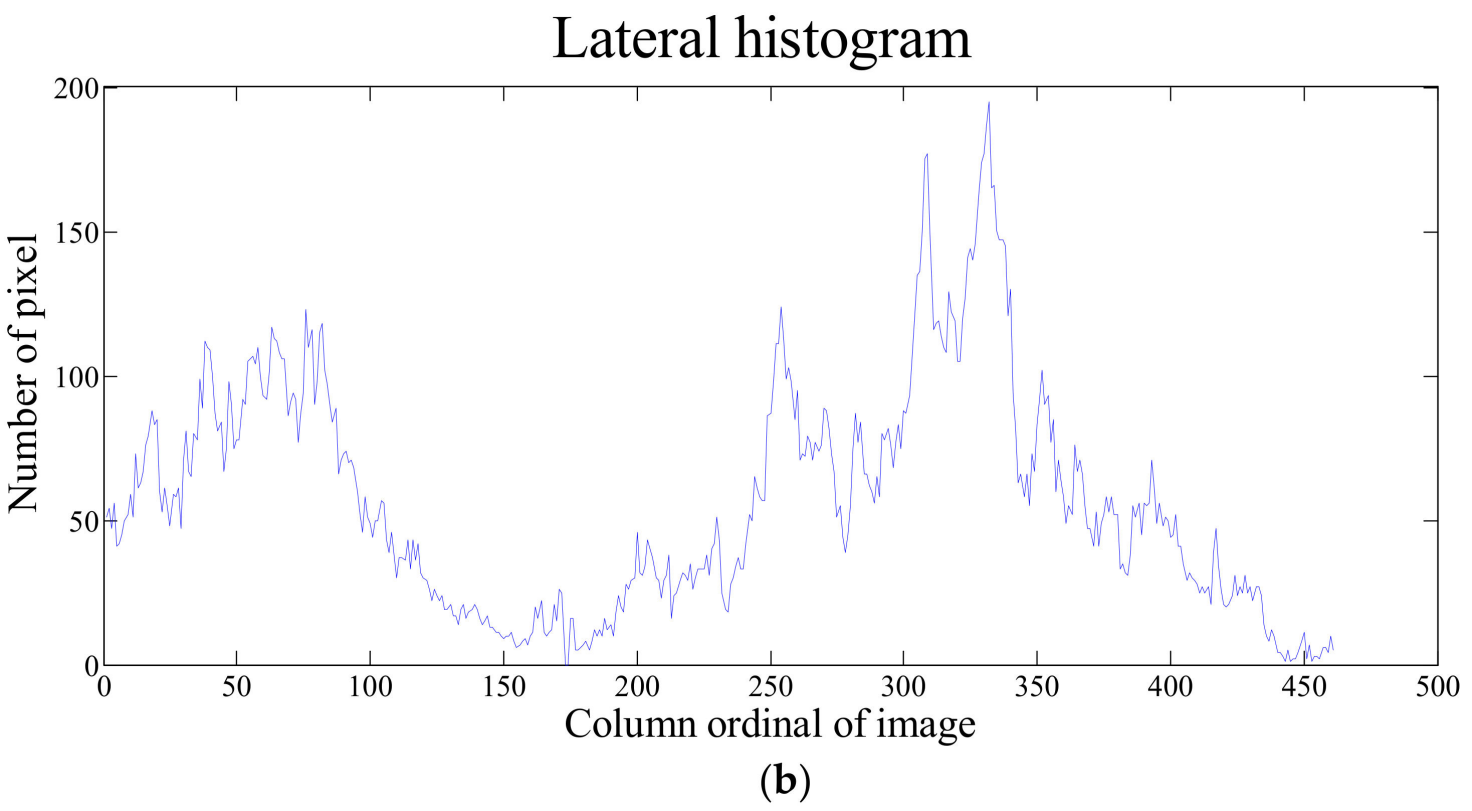
Lateral histogram of field images: (**a**) Pixel lateral histogram of Figure 7(a4); (**b**) Pixel lateral histogram of Figure 7(b4).

The extraction algorithm (proposed in Section 2.3) was used to process the images, (a4 and b4) shown in Figure 7, and extract the corresponding pixel lateral. The number of pixels representing the green vegetation were counted to obtain the lateral histogram, as shown in Figure 8. 

The percentage of inter-row weed distribution was calculated using Equation (8). Then, the application rate of herbicides was determined accordingly. The percentages were 19.42% and 13.06% for the images shown in Figure 7(a4,b4). 

### 3.3. Field Validation of Weed Mapping and VRHS System 

#### 3.3.1. Real-Time Weed Mapping 

In the field testing experiments, the speed of the spraying tractor was 2.5 m/s. The distance from the image weeds to the nozzle was 5 m, which can be adjusted by changing the imaging sensor field of view mounted on top of the tractor cab (Figure 1). Thus, for realizing the real-time spraying, the response time of whole VRHS system must be under 2 s. 

Overall, for 25 experiments, the average processing time was 0.2 s. The machine cycle of the DSP chip (TMS320f2812) was 0.08 μs, so according to the control program, the time that the DSP used from receiving the signal to sending out control signal was about 1.2 μs. The processing time was 0.9 s for the chip to send out the control signal for spraying the herbicide. The signals were used to control the solenoid valve, so the theoretical execution time of the VRHS control system was nearly 1 s. The response time of the overall VRHS system was about 1.2 s (1 s + 0.2 s), which can accommodate the request of real-time processing. 

In the field experiment, the response time from the system start to nozzles spraying for overall system was tested using chronograph (PS-1003, ZhuiRi, ShangHai, China) based on five treatments (different times between 25–27 May 2017), each of which was repeated five times. The results are shown in Table 2.

From Table 2, it is evident that the actual response time of VRHS was longer than the theoretical value. This was mainly because the actual operation had some unpredictable factors, including friction and human factors. However, the actual test time in the field experiments was 1.562 s, which was under the required time of 2 s. Thus, the VRHS system can realize real-time operations.

#### 3.3.2. Variable Rate Spraying 

According to the flow rate parameters of the nozzles, the theoretical values of herbicide application rate under seven combinations of opening and closure of solenoid valves at five pressure levels were shown in Table 3.

In Table 3, the herbicide application rates under spraying mode-3 and -4 were almost equal under the same pressure. The spraying data of mode-4 were not collected or processed in the subsequent process. Therefore, the seven modes were reduced to six modes for the study. 

The DSP controller detected the instantaneous flow rate of total pipelines at five pressure levels, respectively. The flow rate meter in front of each nozzle detected the instantaneous flow rate of each branch. Combining the two sets of data, 10 values were acquired for each spraying mode and the average was taken as the final flow rate of the nozzles under six spraying modes. The results are shown in Table 4. 

It can be seen from Table 4 that the instantaneous flow rate cannot be detected due to low pressure (200 kpa) and small nozzle radius under mode-1. Also, the minimal flow rate was not detected by the flow meter. Thus, improvements could be made by replacing the flow rate meter in the future. 

**Figure 9:**
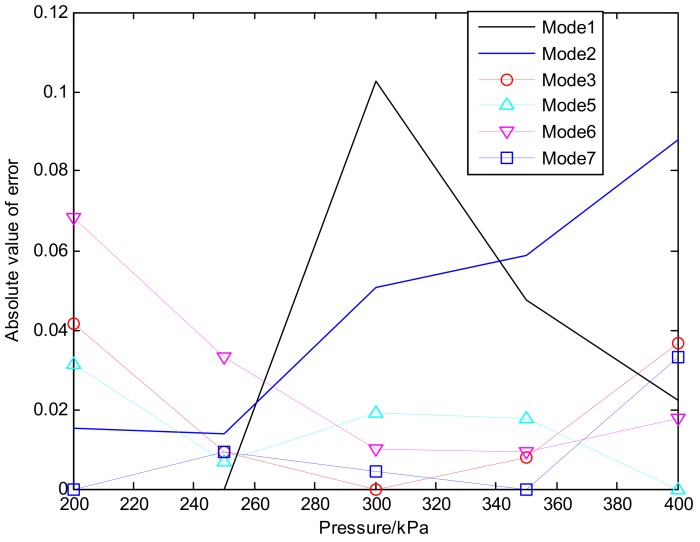
Absolute error between the actual flow rate and the theoretical flow rate.

According to the data of Table 3 and Table 4, the absolute error between the actual flow rate and the theoretical flow rate can be calculated and the corresponding error curve was shown in Figure 9. It was shown that under other four pressures (except 200 kpa, i.e., mode-1), the error between the actual flow rate and the theoretical flow rate was less than 10% (Table 3 and Table 4). Under mode-5, -6 and -7, when the pressure was 200–300 kpa the error was still less than 10%, but the actual value was always smaller than the theoretical value. Experiments were repeated 50 times. When three nozzles were operating, the pressure distributed to each pipeline was compensated to some extent, leading to actual spraying pressure in each pipeline smaller than corresponding theoretical value. When all three pipelines were operating, the pressure in the most distant pipelines achieved the most compensated values. When the pressure of the distributor was over 300 kpa, there would be no fluid returning in the return pipe and all pressure of the system was concentrated in the outlet of the distributor. As a result, the actual pressure of the nozzles would be slightly larger than the measured pressure, and the actual flow rate would be slightly higher than the theoretical value. 

The herbicide application rate for each hectare was calculated using Equation (11):

(11) $$R\;=\;\frac{60,000\times Q}{V\times W}$$

where, *R* is the herbicide application rate for each hectare (L/hm^2^); *Q* is nozzle flow rate (L/min); *V* is speed of the spraying tractor (m/s); *W* is the distance between the nozzles (cm). Using Equation (9), the total herbicide application rate per unit area under each mode was calculated, as shown in Table 5.

In many variable-rate herbicides spraying systems studied so far, the pressure of the outlet was controlled by solenoid valves and a single nozzle was used for spraying one row of crops. According to Table 5, the measuring range of herbicide application rate per unit area (for three nozzles) can be only 84–105.6 L/hm^2^, 153.6–237.6 L/hm^2^ or 220.8–338.4 L/hm^2^, allowing very limited adjustment of application rate. Such a design cannot meet the requirements when the weeds show extremely non-uniform distributions. In contrast, the measuring range of herbicide application rate per unit area was from 84 L/hm^2^ to 674.4 L/hm^2^ using the spraying system described in this study. Overall, the designed system can be applicable for wider range of application rate adjustments that are typical in fields with varied weed distributions.

## 4. Conclusions

In this study, a VRHS system with DSP controller was developed to control the rate of spraying according to real-time weed distribution mapping. The key conclusions of the present study are as follows:

(1) An improved particle swarm optimum algorithm capable of segmenting field images was developed in this study. The algorithm based on lateral histogram for fast extraction of weed maps could successfully extract the weed distribution information. On this basis, the herbicide application rates were determined and transmitted to a computer via a wireless communication module.
(2) A VRHS control system based on DSP processor was developed, with enhanced operating performance due to higher logic operation capacity of the central processor. The spraying part, with a 3—nozzle structure was simple, and the adjustable spraying amount ranges were wide, so the defects of conventional controllers were overcome.
(3) The algorithms (segmentation and weed extraction) proposed in this study were validated in the field. The results showed that the IPSO algorithm was accurate, with reduced error rates from 7.1% to 0.1%, and a processing speed of 0.026 s/frame. In addition, the extraction algorithm based on lateral histogram can extract weed map rapidly.
(4) The integrated variable-rate herbicide spraying system was also validated in a corn field. Experimental results showed that the image processing time of the weed mapping system 0.2 s (was good), and the response time of the overall system was 1.562 s, which meet the real-time process requirements. The adjustable flow rate range was wide, and the herbicide utilization rate was improved.

## Figures and Tables

**Table 1:** Comparison of performance parameters using different segmentation algorithms.

Algorithm	Image Size (Pixels × Pixels)	Threshold Value	Optimization Number	Running Time (s)	Optimization Rate (%)	Error Rate
OTSU	1280 × 1060	38.9	30	0.145	100.0	0.0
PSO	1280 × 1060	36.1	13	0.032	43.3	7.1
IPSO	1280 × 1060	38.6	28	0.026	93.3	0.1

**Table 2:** The response time of overall VRHS system.

Treatment	Response Time (s)					Mean Response Time (s)
	1	2	3	4	5	
1	1.530	1.510	1.550	1.590	1.600	1.556
2	1.560	1.540	1.530	1.570	1.610	1.562
3	1.540	1.550	1.570	1.590	1.580	1.566
4	1.500	1.550	1.600	1.570	1.590	1.562
5	1.590	1.580	1.570	1.540	1.540	1.564

**Table 3:** Theoretical parameters of different types of spraying methods.

Pressure (kpa)	Mode 1	Mode 2	Mode 3	Mode 4	Mode 5	Mode 6	Mode 7
		Flow Rate (L/min)					
200	0.32	0.65	0.96	0.97	1.28	1.61	1.93
250	0.36	0.73	1.08	1.09	1.44	1.81	2.17
300	0.39	0.79	1.18	1.18	1.57	1.97	2.36
350	0.42	0.85	1.28	1.27	1.70	2.13	2.55
400	0.45	0.91	1.36	1.36	1.81	2.27	2.72

**Table 4:** Instantaneous flow rate of six spraying modes in different pressure.

Pressure (kpa)	Mode 1	Mode 2	Mode 3	Mode 5	Mode 6	Mode 7
	Flow Rate (L/min)					
200	0.00	0.64	0.92	1.24	1.50	1.93
250	0.36	0.72	1.09	1.43	1.87	2.15
300	0.35	0.83	1.18	1.54	1.99	2.35
350	0.40	0.90	1.27	1.73	2.15	2.55
400	0.44	0.99	1.41	1.81	2.31	2.81

**Table 5:** Total spraying flow rate of six spraying modes in different pressure.

Pressure (kpa)	Mode 1	Mode 2	Mode 3	Mode 5	Mode 6	Mode 7
	Total Flow Rate (L/hm^2^)					
200	0.0	153.6	220.8	297.6	360.0	463.2
250	86.4	172.8	261.6	343.2	448.8	516.0
300	84.0	199.2	283.2	369.6	477.6	564.0
350	96.0	216.0	304.8	415.2	516.0	612.0
400	105.6	237.6	338.4	434.4	554.4	674.4
